# Particulars of a Case of Extra-Uterine Gestation

**Published:** 1823-04

**Authors:** 


### Art. X.—*Particulars of a Case of Extra-Uterine Gestation.* By the Editors.

Mr. Gunning has very kindly permitted us to give the sketch of an interesting case, which is now under his care at St. George's Hospital. The case will be more fully detailed by that gentleman at some future period; but the following particulars cannot fail of being interesting to our readers.

Elizabeth Atterwell was admitted into St. George's Hospital, on the 5th of March, 1823. She was a married woman, aged twenty-eight, and had been delivered in the year 1815 of her first child, four years after her marriage. The birth of this child took place some weeks earlier than was expected, in consequence of her having experienced a fright. Six years after this, she again suspected herself to be pregnant: she quickened at about five months; but at the end of the seventh month her menses re-appeared, after which she did not again feel the child. At the full period of nine months, labour-pains came on, and continued for four days, when they ceased. From that time there was no alteration in the size of the abdomen, and she experienced no other inconvenience than that which arose from the weight of the tumor. At the natural period after the cessation of the labour-pains, her menses returned, and have continued regularly, with a trifling exception. Suppuration lately took place in the tumor, a little below the umbilicus, and the discharge has become more offensive within these three weeks. The tumor, which at first lay more towards the left side of the abdomen, gradually extended towards the right, accompanied by a sense of throbbing; and the pain has principally been referred to that side. On examination with a probe, bone was readily felt, and the cavity presented to the touch a great irregularity of depth. On the 12th of this month, Mr. Gunning introduced a probe-pointed bistoury into the opening of this tumor, and enlarged it cautiously upwards towards the umbilicus: he then introduced his finger, and removed without difficulty two bones, which, on examination, proved to be two of the vertebræ. A larger portion of bone next presented itself; but it was not loose, and the attempt to move it causing considerable pain, it was judged proper to wait until it should become loosened; the wound being kept dilated by means of the sponge tent.

